# Acceleration of Near-IR Emission through Efficient Surface Passivation in Cd_3_P_2_ Quantum Dots

**DOI:** 10.3390/ma16196346

**Published:** 2023-09-22

**Authors:** Logan Smith, K. Elena Harbison, Benjamin T. Diroll, Igor Fedin

**Affiliations:** 1 Department of Chemistry and Biochemistry, The University of Alabama, Tuscaloosa, AL 35487, USA; 2 Center for Nanoscale Materials, Argonne National Laboratory, 9700 S. Cass Ave, Lemont, IL 60439, USA

**Keywords:** near-IR emitters, II–V quantum dots, time-resolved photoluminescence

## Abstract

Fast near-IR (NIR) emitters are highly valuable in telecommunications and biological imaging. The most established NIR emitters are epitaxially grown In_x_Ga_1−x_As quantum dots (QDs), but epitaxial growth has several disadvantages. Colloidal synthesis is a viable alternative that produces a few NIR-emitting materials, but they suffer from long photoluminescence (PL) times. These long PL times are intrinsic in some NIR materials (PbS, PbSe) but are attributed to emission from bright trapped carrier states in others. We show that Cd_3_P_2_ QDs possess substantial trap emission with radiative times >10^1^ ns. Surface passivation through shell growth or coordination of Lewis acids is shown to accelerate the NIR emission from Cd_3_P_2_ QDs by decreasing the amount of trap emission. This finding brings us one step closer to the application of colloidally synthesized QDs as quantum emitters.

## 1. Introduction

Near-infrared (NIR) radiation (700–1700 nm) enables secure telecommunications, threat detection, and biomedical imaging. The NIR-I window (700–950 nm) covers functional NIR imaging (730 and 850 nm) and the first telecom window (800–900 nm), while the NIR-II window (1000–1700 nm) covers biological tissue imaging and six telecom bands (1260–1675 nm). The S, C, U, and L telecom bands (1460–1675 nm) are particularly valuable owing to the low propagation losses in optical fibers, low dispersion, and the operation of lanthanide-doped amplifiers ca. 1530–1560 nm. Brain tissue is highly transparent in the region from 1100 to 1350 nm [1]. These applications require bright, fast, and durable emitters tunable in the desired wavelength range. Epitaxial III–V semiconductors (e.g., In_x_Ga_1−x_As) are ubiquitous in near-IR emitters and detectors owing to their bandgap tunability, short radiative times, and ease of coupling to resonant cavities. Single In_x_Ga_1−x_As quantum dots (QDs) have shown long coherence times and photon indistinguishability. Epitaxial growth, however, is area scalable, requires sophisticated apparatus, and involves toxic volatile precursors. Moreover, growing two QDs of identical photon energy within the radiative linewidth remains unachievable but highly desirable in quantum communication [2]. Colloidal synthesis, on the other hand, offers a great degree of bandgap tunability, provides substantial control over the QDs surface, and affords gram quantities of the material per synthesis. The most established colloidal NIR QD emitters include lead chalcogenides [3,4], mercury chalcogenides [5,6], and InAs [7,8]. The PL lifetime of PbS and PbSe QDs is in the microsecond range [4,9], which limits their application in quantum emitters. Mercury chalcogenides QDs exhibit fast PL lifetimes; however, the reported examples of bright QDs only cover the region up to 1300 nm or the region up from 1700 nm. Colloidal III–V QDs suffer from surface trap states and anti-site defects, which impair their PL properties [10]. The most perfected colloidal III–V near-IR QDs (InAs/CdSe) show radiative lifetimes of down to 130 ns [7]. Despite the superior performance of these QDs, their method of synthesis is non-trivial: the arsenic precursors, *tris*(trimethylsilyl)arsine and *tris*(trimethylgermanyl)arsine, are not commercially available and must be synthesized in the lab.

In this study, we develop the colloidal chemistry of an overlooked II–V semiconductor cadmium phosphide (Cd_3_P_2_)—an alternative promising near-IR material that offers the necessary bandgap tunability. It is a unique material that bridges III–V and II–VI semiconductors and combines the best of both worlds: near-IR emission with solution processability [11]. The bandgap of bulk Cd_3_P_2_ is 0.55 eV, but QDs can be tuned to absorb and emit in the range from 800 nm to 1550 nm [12,13], which covers NIR I and II windows. NIR-emitting Cd_3_P_2_ QDs are susceptible to Zn_3_P_2_ shell growth [14] and have been successfully incorporated in silica and polystyrene for tissue imaging [15]. Despite these advantages, there are still some drawbacks that make it difficult for Cd_3_P_2_ to compete with developed NIR emitters. The PL lifetime of Cd_3_P_2_ QDs is hundreds of nanoseconds, which is longer than predicted for the dielectric constant and the bandgap of Cd_3_P_2_ QDs [16]. Long PL dynamics is an obstacle to using Cd_3_P_2_ QDs as on-demand single-photon emitters. Long PL dynamics has been attributed to emission from bright trap states [17], and undesired carrier trapping can be mitigated through efficient surface passivation [18]. Here, we show that effective surface passivation techniques result in the reduction in trap emission from Cd_3_P_2_ QDs, leading to the observed acceleration of PL dynamics coupled with higher percentages of band-edge emission.

## 2. Materials and Methods

We synthesized Cd_3_P_2_ QDs via a modified recipe previously published by A. Eychmüller et al. [12] and X. Peng et al. [13]. Namely, 0.50 mmol *tris*-(trimethylsilyl) phosphine was injected into a hot solution of 1.50 mmol cadmium oleate in 1-octadecene at 195–215 °C and reacted for 40 s–6 min. The size of the QDs was tuned by varying the amount of free oleic acid and the injection temperature: larger amounts of free oleic acid resulted in the nucleation of larger QDs (up to the solubility limit); a combination of lower injection temperatures (down to 195 °C) and longer reaction times (up to 6 min) produced larger QDs. Powder X-ray diffraction (XRD) of a drop-casted layer of washed Cd_3_P_2_ QDs was measured on a Bruker D8 Discover diffractometer. To passivate the surface of the Cd_3_P_2_ QDs, we treated them with selenium in oleylamine at 140 °C, followed by a coating with a layer of Cd acetate at room temperature [19]. We confirmed the deposition of a layer of CdSe with ICP OES. To synthesize Cd_3_P_2_ QDs passivated with CdI_2_, we injected 0.10 mmol *tris*-(trimethylsilyl) phosphine into a solution of 0.30 mmol cadmium iodide solubilized with trioctylphosphine in octadecene at 215 °C. Finally, to passivate the surface of Cd_3_P_2_ QDs with iodocadmate, we performed a ligand exchange of oleate-capped Cd_3_P_2_ QDs in toluene into a 0.10 M solution of NH_4_CdI_3_ in formamide [20]. To remove excess ligands, we precipitated the QDs with acetonitrile and toluene and redispersed them in pure formamide or *N*-methylformamide. The steady-state PL spectra of the QDs emitting up to 900 nm were measured in toluene on a Horiba Fluoromax+ spectrofluorometer (Horiba, Kyoto, Japan) with the excitation wavelength of 475 nm and no bandpass filter on the detector. The steady-state PL spectra of the QDs emitting above 1000 nm were measured in tetrachloroethylene on a Horiba Fluoromax+ spectrofluorometer with an IGA detector. A 715 nm bandpass filter was placed before the IGA detector to filter out the excitation wavelength. The PL QY of the QDs emitting above 1000 nm was measured against a standard of commercial PbS QDs with a QY of 30%. Time-resolved PL of the QDs emitting up to 900 nm were measured in toluene on a Horiba Delta Pro (Horiba, Kyoto, Japan) using a 629 nm laser diode with a typical pulse width of 50 ps and a frequency between 250 and 500 kHz. A 715 nm long-pass filter was placed before the detector. Time-resolved PL of the QDs emitting at 1000–1250 nm were measured using a superconducting nanowire array synchronized with a 705 nm pulsed excitation. The frequency of the excitation source ranged between 500 kHz and 1 MHz. The pump intensity was low enough to eliminate the possibility of multiexciton formation, which we confirmed by measuring PL dynamics at a series of excitation powers. Because the resulting traces overlay perfectly, Auger recombination dynamics from multiexcitons were absent.

## 3. Results and Discussion

### 3.1. Synthesis and Surface Passivation

**Figure 1:**
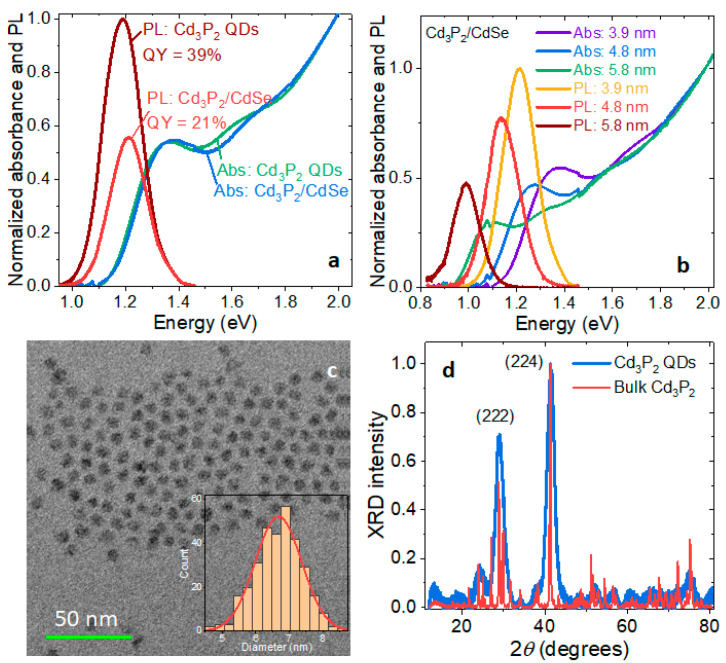
(**a**) Normalized absorption and photoluminescence (PL) spectra of 3.9 nm Cd_3_P_2_ and Cd_3_P_2_/CdSe quantum dots (QDs) in toluene. The percentages show the PL quantum yields. (**b**) Normalized absorption and PL spectra of Cd_3_P_2_/CdSe QDs of three different sizes. The numbers indicate the diameters of the corresponding Cd_3_P_2_ cores. (**c**) Transmission electron micrograph (TEM) of the reddest Cd_3_P_2_/CdSe QDs shown in (**b**). The inset is a distribution histogram of the QDs by their diameter. (**d**) Powder XRD spectra of a layer of oleate-capped Cd_3_P_2_ QDs and a layer of bulk Cd_3_P_2_ powder. Miller indices of the tetragonal Cd_3_P_2_ crystal structure are shown for the two strongest peaks of the QD spectrum.

A typical batch of oleate-capped Cd_3_P_2_ QDs showed an emission peak at 1.19 eV with the full width at half-maximum (fwhm) of 170 meV and a PL QY of 39% (Figure 1a). The powder XRD pattern of these Cd_3_P_2_ QDs showed two strong peaks corresponding to the (222) and (224) families of planes of the tetragonal crystal structure (Figure 1d). These QDs were stable under dry nitrogen but degraded in air over two days. The treatment with CdSe resulted in a slight blue shift of the PL to 1.21 eV with the fwhm of 164 meV, a decrease in the PL QY to 21%, and a prolonged stability of the QDs under ambient conditions. The small blue shift in the absorption and PL spectra is a result of a gentle etching of Cd_3_P_2_ with oleylamine during the treatment with Se [21]. Larger Cd_3_P_2_/CdSe QDs showed lower PL QYs (16% and 8.4%, Figure 1b), which is consistent with the previously reported results for other near-IR emitters [7,22]. The reduction in the PL QY comes from the lattice mismatch between the tetragonal Cd_3_P_2_ and zincblende CdSe crystal structures. Core-shell Cd_3_P_2_ were noticeably larger than the parent Cd_3_P_2_ QDs in TEM (e.g., 6.7 nm vs. 5.8 nm; Figure 1c), which, together with ICP measurements (Table S1), confirmed the growth of a CdSe shell. Larger Cd_3_P_2_/CdSe QDs appeared faceted in TEM (Figure 1c), consistent with previous reports [13] and in accord with the tetragonal crystal structure. Cd_3_P_2_ QDs synthesized from CdI_2_ were smaller in size, with their PL peak not exceeding 900 nm.

### 3.2. Time-Resolved Spectroscopy

Measured PL dynamics of Cd_3_P_2_ QDs of various surface passivation showed a multiexponential decay. We fit the PL decay as multiexponential with the minimal number of components: I=Ase−tτs+Aie−tτi+ALe−tτL, where *τ*_S_, *τ*_i_, and *τ*_L_ are the short, intermediate, and long components, respectively. The fraction of all light that is emitted through a particular channel ($f_{x}$) is calculated as:

(1) $$f_{x}=\frac{A_{x}\tau_{x}}{A_{s}\tau_{s}+A_{i}\tau_{i}+A_{L}\tau_{L}}$$

**Figure 2:**
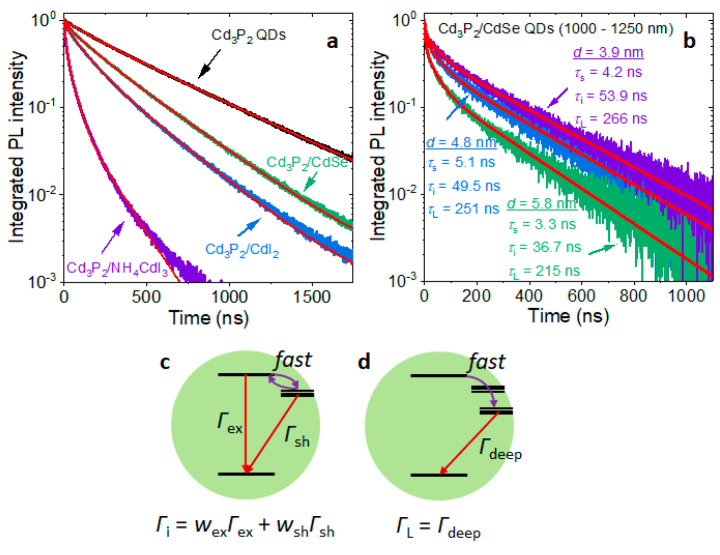
PL dynamics of bare and passivated Cd_3_P_2_ QDs. (**a**) Time-resolved PL of Cd_3_P_2_, Cd_3_P_2_/CdSe, Cd_3_P_2_/CdI_2_, and Cd_3_P_2_/NH_4_CdI_3_ QDs excited with a 629 nm pulsed laser diode and emitting ca. 850 nm, fitted with the minimal number of multiexponentials. (**b**) Time-resolved PL of Cd_3_P_2_/CdSe QDs excited with a 705 nm laser with the emission peaks at 1000–1250 nm (the QDs shown in Figure 1b). (**c**) In Cd_3_P_2_ QDs with only shallow traps, the excitation is delocalized between the band edge and trap states. (**d**) In Cd_3_P_2_ QDs with deep traps, the excitation localizes in a trap state.

PL dynamics of oleate-capped Cd_3_P_2_ QDs showed a relatively slow biexponential decay with the long component of 490 ns (Figure 2a, Table 1), which is comparable to the previously reported values in InAs/CdSe QDs [7]. Cd_3_P_2_/CdSe core-shell QDs routinely showed faster emission dynamics compared to oleate-capped Cd_3_P_2_ QDs. Analysis shows the appearance of a fast 21.2 ns component and acceleration of the slow component (to 327 ns). The fraction of the intermediate component increased from 6.0 to 13.8% in Cd_3_P_2_/CdSe compared to oleate-capped Cd_3_P_2_ QDs. Larger Cd_3_P_2_/CdSe QDs generally showed faster PL dynamics, which accelerated with the increasing size of the QDs (Figure 1b and Table 2). This observation is in contrast with previously reported data for InAs/CdSe QDs [6].

Surface passivation with CdI_2_ showed a greater acceleration of PL dynamics mostly due to the increase in the fraction of the intermediate component (116 ns) to 28.5%. Finally, Cd_3_P_2_ QDs ligand-exchanged with NH_4_CdI_3_ and dispersed in formamide showed substantially faster PL dynamics through all channels, which is the effect of the negatively charged surface ligand layer. This observation is consistent with InAs/CdSe QDs showing faster PL dynamics in water compared to organic solvents [7]. Moreover, the majority of all light emitted from Cd_3_P_2_/NH_4_CdI_3_ QDs came from the intermediate emission channel (42.6 ns). The appearance of the fast component (7.5 ns) decreased the QY of the QDs by an order of magnitude compared to the original oleate-capped QDs, which is consistent with the large value of the corresponding pre-exponential, A_S_ = 0.458. The origin of the 7.5 ns component is unclear; nanosecond dynamics is often attributed to QD charging or carrier trapping. Charging would result in Auger recombination, which occurs on the time scale of 10–100 ps [23], which would not be resolvable in our measurements. Carrier trapping, on the other hand, can occur on the 10 ps [24] to 10 ns [25] scale, which can explain the observed fast dynamics.

### 3.3. Surface Trap States

The measured multiexponential PL dynamics indicates multiple emission channels in Cd_3_P_2_ QDs—we have a heterogeneous ensemble of emitters. Given that the dielectric constant of Cd_3_P_2_ (5.8) [12] is very close to that of CdSe (5.9) [26], QDs of both materials of similar bandgap are expected to have comparable band-edge emission times (20 ns) [16]. We expect band-edge emission from narrower-gap Cd_3_P_2_ QDs to be slower [16] but still within the 10^1^ ns range. The measured long components in the PL dynamics, therefore, do not correspond to band-edge emission but likely correspond to surface trap emission. It was shown previously that while uncoordinated Cd atoms in CdSe QDs do not act as electron traps, unpassivated lone pairs of surface Se atoms can act as hole traps [18]. By analogy with CdSe, we hypothesize the long hundred-nanosecond PL in Cd_3_P_2_ originates from the recombination of band-edge electrons with holes trapped at surface P atoms. The intermediate component of the PL dynamics of Cd_3_P_2_ and Cd_3_P_2_/CdI_2_ QDs is too long to be pure band-edge emission. We hypothesize that we have a heterogeneous ensemble of QDs with only shallow traps (Figure 2c) and QDs with both deep and shallow traps (Figure 2d). Carriers relax into the trap states on the picosecond time scale, which we do not resolve in our transient PL measurements. In QDs with shallow trap states, the excitation delocalizes between a trap state and the band-edge exciton. Therefore, the radiative recombination rate in these QDs is a weighted average of the band-edge exciton recombination rate (Γ_ex_) and the shallow-trap radiative recombination rate (Γ_sh_) (Figure 2a). In QDs with deep trap states, the radiative recombination comes only from the trap states. The analysis of PL dynamics suggests that, in a sample of oleate-capped Cd_3_P_2_ QDs, 6.0% of Cd_3_P_2_ QDs have only shallow traps and 94% of QDs have deep traps (Table 1). The acceleration and increase in the weight of the intermediate component in Cd_3_P_2_/CdSe QDs suggests that overgrowth of a CdSe shell removes some shallow and deep trap states. Surface passivation with CdI_2_ eliminates an even greater fraction of trap states. By analogy with surface passivation in CdSe QDs [18], when CdI_2_ coordinates to the lone pair of electrons of a surface P atom, it lowers its energy below the level of the 1S_hole_ state, eliminating the hole trap state.

### 3.4. Stokes Shifts

**Figure 3:**
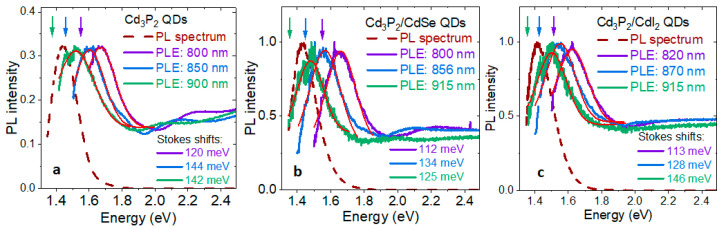
Photoluminescence excitation (PLE) spectra of (**a**) oleate-capped Cd_3_P_2_ QDs, (**b**) Cd_3_P_2_/CdSe QDs, and (**c**) Cd_3_P_2_/CdI_2_ QDs, emitting ca. 850 nm. The arrows point to the monitored emission energies within the PL band of each sample.

To determine if the PL of Cd_3_P_2_ QDs originates from trap states, we measured the Stokes shifts in the photoluminescence excitation (PLE) spectra of oleate-capped Cd_3_P_2_, Cd_3_P_2_/CdSe, and Cd_3_P_2_/CdI_2_ QDs. For each sample, we monitored the PL at three different energies within the emission band and collected the excitation spectra (Figure 3). PLE spectroscopy produces more accurate values for the Stokes shifts than a combination of absorption and PL spectroscopies because not only are the spectral features sharper in PLE than in absorption spectra, but the peak in the PLE spectrum is also the absorption peak of the subset of the ensemble that emits at the monitored wavelength (energy). 

The measured Stokes shifts were generally comparable to the previously reported ones for Cd_3_P_2_ [12,13]. In all samples, the bluest subensembles showed the smallest Stokes shifts (of 112–120 meV). In Cd_3_P_2_ and Cd_3_P_2_/CdSe QDs, the middle subensembles showed the greatest Stokes shifts (144 meV and 142 meV). In Cd_3_P_2_/CdI_2_ QDs, the trend was monotonous, and the greatest Stokes shift (146 meV) was observed in the reddest subensemble. This trend is the opposite of what was reported for CdSe QDs [27], where the large Stokes shifts in small CdSe QDs were explained by the combination of large exciton fine structure splitting and exciton–phonon coupling. The measured Stokes shifts for Cd_3_P_2_ are substantially higher than those reported for CdSe QDs (26–80 meV). One reason is that the energy of the LO phonon of Cd_3_P_2_ (51 meV) is substantially higher than that of CdSe (26.5 meV), but it does not fully account for the observed difference. The Stokes shifts in Cd_3_P_2_ QDs are comparable to those in HF-treated InP QDs (150–200 meV) [10], where they were attributed to hole trapping and coupling of the exciton to the LO phonon. Therefore, the Stokes shifts we observed in Cd_3_P_2_ QDs are likely due to hole trapping at surface P atoms [28] and exciton–LO-phonon coupling. Larger QDs have a greater number of hole traps at the surface, hence the larger Stokes shifts. As we transition from oleate-capped Cd_3_P_2_ QDs to Cd_3_P_2_/CdSe and Cd_3_P_2_/CdI_2_ QDs, there is a reduction in the Stokes shift by up to 17 meV, except for the reddest subensemble of Cd_3_P_2_/CdI_2_ QDs, which can be attributed to the elimination of some trap states because of surface passivation.

## 4. Conclusions

Surface passivation through the overgrowth of a CdSe shell, coordination of CdI_2_ Lewis acid, or ligand exchange with NH_4_CdI_3_ accelerates the PL dynamics of oleate-capped Cd_3_P_2_ QDs. This acceleration comes from the acceleration of the long component and the increase in the weight of the intermediate component in the multiexponential dynamics of the QDs. We attribute the long (10^2^ ns) PL dynamics in Cd_3_P_2_ to hole trapping at the surface P atoms, which act as shallow and deep trap states. This attribution is consistent with the large Stokes shifts (112–146 meV) measured in the PLE spectra of Cd_3_P_2_ QDs. We explain the observed acceleration of the PL dynamics as a result of surface passivation leading to the reduction in the number of trap states due to the coordination of Cd^II^ of CdI_2_ or CdSe to the surface P atoms, which is in accord with the small reduction in the Stokes shifts. These methods of surface passivation render Cd_3_P_2_ QDs capable of fast, tunable emission with high solution processability, expanding many of the highlighted photophysical properties of colloidal QDs in the NIR region. Ultimately, these results bring the incorporation of quantum emission closer to improved telecommunications, biological imaging, and threat detection.

## Figures and Tables

**Table 1:** Analysis of the multiexponential PL dynamics shown in Figure 2a. The short (*τ*_S_), intermediate (*τ*_i_), and long (*τ*_L_) components are listed together with the fraction of all light that is emitted through the corresponding channel.

Material	*τ*_S_ (ns)	*τ*_i_ (ns)	*τ*_L_ (ns)
Cd_3_P_2_		162 ns (6.0%)	491 ns (94.0%)
Cd_3_P_2_/CdSe	21.2 ns (0.7%)	139 ns (13.8%)	327 ns (85.4%)
Cd_3_P_2_/CdI_2_	16.9 ns (1.1%)	116 ns (28.5%)	291 ns (70.4%)
Cd_3_P_2_/NH_4_CdI_3_	7.5 ns (8.7%)	42.6 ns (52.4%)	152 ns (38.8%)

**Table 2:** Analysis of the multiexponential PL dynamics shown in Figure 2b. The short (*τ*_S_), intermediate (*τ*_i_), and long (*τ*_L_) components are listed together with the fraction of all light that is emitted through the corresponding channel.

QD Diameter	*τ*_S_ (ns)	*τ*_i_ (ns)	*τ*_L_ (ns)
3.9 nm	4.2 ns (0.9%)	53.9 ns (12.2%)	266 ns (86.9%)
4.8 nm	5.1 ns (1.8%)	49.5 ns (15.6%)	251 ns (82.6%)
5.8 nm	3.3 ns (2.6%)	36.7 ns (21.1%)	215 ns (76.3%)

## Data Availability

Not applicable.

## Supplementary Materials

The following supporting information can be downloaded at: https://www.mdpi.com/article/10.3390/ma16196346/s1, Figure S1: TEM image of 5.8 nm Cd_3_P_2_ QDs emitting at 1250 nm (the reddest sample in Figure 1b); Figure S2: QY measurement of a sample of Cd_3_P_2_ QDs by comparison with a sample of PbS QDs with the QY of 30%; Figure S3: PL dynamics of bare and passivated Cd_3_P_2_. (a) First 100 ns of time-resolved PL of Cd_3_P_2_, Cd_3_P_2_/CdSe, Cd_3_P_2_/CdI_2_, and Cd_3_P_2_/NH_4_CdI_3_ QDs excited with a 629 nm pulsed laser diode and emitting ca. 850 nm fitted with the minimal number of multiexponentials. (b) Time-resolved PL of Cd_3_P_2_, Cd_3_P_2_/CdSe, Cd_3_P_2_/CdI_2_, and Cd_3_P_2_/NH_4_CdI_3_ QDs excited with a 629 nm pulsed laser diode and emitting ca. 850 nm fitted with the minimal number of multiexponentials. (c) First 100 ns of time-resolved PL of Cd_3_P_2_/CdSe QDs excited with a 705 nm laser with the emission peaks 1000–1250 nm (the QDs shown in Figure 1b). (d) Time-resolved PL of Cd_3_P_2_/CdSe QDs excited with a 705 nm laser with the emission peaks 1000–1250 nm (the QDs shown in Figure 1b); Table S1: Atomic compositions of Cd_3_P_2_ and Cd_3_P_2_/CdSe QDs measured by ICP OES and referred to P (2.00).

## References

[1] Feng Z., Tang T., Wu T., Yu X., Zhang Y., Wang M., Zheng J., Ying Y., Chen S., Zhou J. (2021). Perfecting and extending the near-infrared imaging window. Light Sci. Appl..

[2] Senellart P., Solomon G., White A. (2017). High-performance semiconductor quantum-dot single-photon sources. Nat. Nanotechnol..

[3] Moreels I., Justo Y., De Geyter B., Haustraete K., Martins J.C., Hens Z. (2011). Size-Tunable, Bright, and Stable PbS Quantum Dots: A Surface Chemistry Study. ACS Nano.

[4] Bae W.K., Joo J., Padilha L.A., Won J., Lee D.C., Lin Q., Koh W.-k., Luo H., Klimov V.I., Pietryga J.M. (2012). Highly Effective Surface Passivation of PbSe Quantum Dots through Reaction with Molecular Chlorine. J. Am. Chem. Soc..

[5] Sayevich V., Robinson Z.L., Kim Y., Kozlov O.V., Jung H., Nakotte T., Park Y.-S., Klimov V.I. (2021). Highly versatile near-infrared emitters based on an atomically defined HgS interlayer embedded into a CdSe/CdS quantum dot. Nat. Nanotechnol..

[6] Kamath A., Schaller R.D., Guyot-Sionnest P. (2023). Bright Fluorophores in the Second Near-Infrared Window: HgSe/CdSe Quantum Dots. J. Am. Chem. Soc..

[7] Franke D., Harris D.K., Chen O., Bruns O.T., Carr J.A., Wilson M.W.B., Bawendi M.G. (2016). Continuous injection synthesis of indium arsenide quantum dots emissive in the short-wavelength infrared. Nat. Commun..

[8] Ginterseder M., Franke D., Perkinson C.F., Wang L., Hansen E.C., Bawendi M.G. (2020). Scalable Synthesis of InAs Quantum Dots Mediated through Indium Redox Chemistry. J. Am. Chem. Soc..

[9] Cheng C., Li J., Cheng X. (2017). Photoluminescence lifetime and absorption spectrum of PbS nanocrystal quantum dots. J. Lumin..

[10] Janke E.M., Williams N.E., She C., Zherebetskyy D., Hudson M.H., Wang L., Gosztola D.J., Schaller R.D., Lee B., Sun C. (2018). Origin of Broad Emission Spectra in InP Quantum Dots: Contributions from Structural and Electronic Disorder. J. Am. Chem. Soc..

[11] Glassy B.A., Cossairt B.M. (2017). II3V2 (II: Zn, Cd; V: P, As) Semiconductors: From Bulk Solids to Colloidal Nanocrystals. Small.

[12] Miao S., Hickey S.G., Rellinghaus B., Waurisch C., Eychmüller A. (2010). Synthesis and Characterization of Cadmium Phosphide Quantum Dots Emitting in the Visible Red to Near-Infrared. J. Am. Chem. Soc..

[13] Xie R., Zhang J., Zhao F., Yang W., Peng X. (2010). Synthesis of Monodisperse, Highly Emissive, and Size-Tunable Cd_3_P_2_ Nanocrystals. Chem. Mater..

[14] McVey B.F.P., Swain R.A., Lagarde D., Ojo W.-S., Bakkouche K., Marcelot C., Warot B., Tison Y., Martinez H., Chaudret B. (2022). Cd_3_P_2_/Zn_3_P_2_ Core-Shell Nanocrystals: Synthesis and Optical Properties. Nanomaterials.

[15] Ding L., He S., Chen D., Huang M., Xu J., Hickey S.G., Eychmüller A., Yu S.-H., Miao S. (2014). Encapsulated Cd_3_P_2_ quantum dots emitting from the visible to the near infrared for bio-labelling applications. CrystEngComm.

[16] Sercel P.C., Efros A.L. (2018). Band-Edge Exciton in CdSe and Other II–VI and III–V Compound Semiconductor Nanocrystals—Revisited. Nano Lett..

[17] Trimpl M.J., Wright A.D., Schutt K., Buizza L.R.V., Wang Z., Johnston M.B., Snaith H.J., Müller-Buschbaum P., Herz L.M. (2020). Charge-Carrier Trapping and Radiative Recombination in Metal Halide Perovskite Semiconductors. Adv. Funct. Mater..

[18] Houtepen A.J., Hens Z., Owen J.S., Infante I. (2017). On the Origin of Surface Traps in Colloidal II–VI Semiconductor Nanocrystals. Chem. Mater..

[19] Gao Y., Peng X. (2015). Photogenerated Excitons in Plain Core CdSe Nanocrystals with Unity Radiative Decay in Single Channel: The Effects of Surface and Ligands. J. Am. Chem. Soc..

[20] Zhang H., Kurley J.M., Russell J.C., Jang J., Talapin D.V. (2016). Solution-Processed, Ultrathin Solar Cells from CdCl_3_^−^-Capped CdTe Nanocrystals: The Multiple Roles of CdCl_3_^−^ Ligands. J. Am. Chem. Soc..

[21] Salzmann B.B.V., Vliem J.F., Maaskant D.N., Post L.C., Li C., Bals S., Vanmaekelbergh D. (2021). From CdSe Nanoplatelets to Quantum Rings by Thermochemical Edge Reconfiguration. Chem. Mater..

[22] Harris D.K., Allen P.M., Han H.-S., Walker B.J., Lee J., Bawendi M.G. (2011). Synthesis of Cadmium Arsenide Quantum Dots Luminescent in the Infrared. J. Am. Chem. Soc..

[23] Robel I., Gresback R., Kortshagen U., Schaller R.D., Klimov V.I. (2009). Universal Size-Dependent Trend in Auger Recombination in Direct-Gap and Indirect-Gap Semiconductor Nanocrystals. Phys. Rev. Lett..

[24] Sung Y.M., Kim T.-G., Yun D.-J., Lim M., Ko D.-S., Jung C., Won N., Park S., Jeon W.S., Lee H.S. (2021). Increasing the Energy Gap between Band-Edge and Trap States Slows Down Picosecond Carrier Trapping in Highly Luminescent InP/ZnSe/ZnS Quantum Dots. Small.

[25] Du J., Singh R., Fedin I., Fuhr A.S., Klimov V.I. (2020). Spectroscopic insights into high defect tolerance of Zn:CuInSe_2_ quantum-dot-sensitized solar cells. Nat. Energy.

[26] Ninomiya S., Adachi S. (1995). Optical properties of cubic and hexagonal CdSe. J. Appl. Phys..

[27] Liptay T.J., Marshall L.F., Rao P.S., Ram R.J., Bawendi M.G. (2007). Anomalous Stokes shift in CdSe nanocrystals. Phys. Rev. B.

[28] Kornowski A., Eichberger R., Giersig M., Weller H., Eychmüller A. (1996). Preparation and Photophysics of Strongly Luminescing Cd_3_P_2_ Quantum Dots. J. Phys. Chem..

