# Supplementary material for: Acceleration of Near-IR Emission through Efficient Surface Passivation in Cd3P2 Quantum Dots
Source: Materials (Basel). 2023 Sep 22;16(19):6346. doi: 10.3390/ma16196346 (PMC10573561; doi:10.3390/ma16196346)
Supplement: Supplementary file 1 [file materials-16-06346-s001.zip › materials-2615215-supplementary.pdf]

Supporting Information

Acceleration of Near-IR Emission through Efficient Surface Passivation in Cd_3P_2 Quantum Dots

Logan Smith [1], K. Elena Harbison [1], Benjamin T. Diroll [2], and Igor Fedin [1]*

1. Department of Chemistry and Biochemistry, The University of Alabama, Tuscaloosa, AL, 35487, United States

2. Center for Nanoscale Materials, Argonne National Laboratory, 9700 S. Cass Ave, Lemont, IL, 60439, United States

E-mail: ifedin@ua.edu

Chemicals

Selenium (Se, 99.99%), cadmium oxide (CdO, ≥ 99.99%), oleylamine (70%), tri-n-butylphosphine (TBP, 99%), and 1-octadecene (ODE, 90%) were purchased from Sigma-Aldrich. Oleic acid (OA, 90%), zinc acetate dihydrate (≥98%), and cadmium acetate dihydrate (≥98%) were purchased from Alfa Aesar. Cadmium iodide (anhydrous, 99.99%), *tris*(trimethylsilyl)phosphine (TMS_3P, 98%) and tri-n-octylphosphine (TOP, 97%) were purchased from Strem. All chemicals were used without any purification.

Synthesis of Nanomaterials

Synthesis of Cd_3P_2 QDs. 192.6 mg (1.50 mmol) CdO was added to a 100 ml round bottom flask. 20 ml of ODE were used as the solvent. The amount of oleic acid present initially in the reaction flask varied depending on the desired emission wavelength. For example, for QDs emitting ~1300 nm, 6.5 mmol of oleic acid was used. The reaction was degassed under vacuum while heating to 110°C for 10 min. After 10 min, the reaction flask was placed under N_2 and allowed to heat to 210°C. At 210°C, a solution of 0.145 ml (0.50 mmol) TMS_3P in 2.5 ml dried ODE was rapidly injected into the reaction flask. A total of 45 s was allowed to elapse before removing the reaction flask from heat. The reaction flask was allowed to cool to room temperature before working up the product in the glovebox. The black solution in the reaction flask was separated into two 50 ml centrifuge tubes. About 5 ml of dried toluene were added to each. Dried ethanol was used as the antisolvent. Once crashed out of solution, the QDs were dried under vacuum. Once dry, 1 ml of dried toluene was added to the QDs. The vial containing the solution of QDs was stored in the glovebox.

Synthesis of Cd_3P_2/CdSe QDs. 20 mg Cd_3P_2 QDs in a small volume of toluene was dissolved in 5.0 ml ODE in a 25 ml round bottom flask until. 0.07 ml of 0.1 M Se solution in OAm and 0.08 ml OAm was added to the reaction flask. The reaction flask was placed under vacuum and degassed at room temperature until all toluene was boiled out. The reaction flask was then placed under N_2 and allowed to heat to 140°C over 3 min. The temperature was then held at 140°C for 7 min. After 7 min had elapsed, 0.2 ml TBP was rapidly injected into the reaction flask to react with any remaining Se. The temperature was then increased to 220°C. Once the temperature reached 220°C, the reaction flask was removed from heat and allowed to cool to 80°C. At ~80°C, 12 mg of cadmium acetate dihydrate was added to the reaction flask. The reaction flask was allowed to cool to room temperature and allowed to stir while remaining under N_2 for 10 min. The reaction flask was moved into the glovebox where it was worked up using toluene and ethanol as the antisolvent. The QDs were redispersed in anhydrous and stored in the glove box.

Synthesis of Cd_3P_2/CdI_2 QDs. 550 mg (1.5 mmol) CdI_2 were added to a 50 ml round bottom flask. 6.5 ml of TOP were used as the solvent. 1.5 mmol of oleic acid were added to the flask. The

reaction vessel was degassed under vacuum while heating to 110°C for 10 min. After 10 min, the reaction flask was placed under N_2 and allowed to heat to 215°C. At 215°C, a solution of (0.145 ml) 0.5 mmol TMS_3P in 2.5 ml dried ODE was rapidly injected into the reaction flask. A total of 45 s was allowed to elapse before removing the reaction flask from heat. The reaction flask was allowed to cool to room temperature before working up the product in the glovebox. The black solution in the reaction flask was separated into two 50 ml centrifuge tubes. About 5 ml of dried toluene were added to each. Dried ethanol was used as the antisolvent. Once crashed out of solution, the QDs were dried under vacuum. Once dry, 1 ml of dried toluene was added to the QDs. The vial containing the solution of QDs was stored in the glovebox.

Ligand exchange. 0.100 M NH_4CdI_3 in formamide (FA) was prepared by dissolving 366 mg (1.00 mmol) CdI_2 and 145 mg (1.00 mmol) NH_4I in FA and diluting the solution to 10.0 ml. 40 mg of oleate-capped Cd_3P_2 QDs in toluene was purified from organics by precipitation with ethanol and redispersion in toluene twice. The resulting sample of Cd_3P_2 QDs was diluted with toluene to 5 ml and stirred with 1.0 ml of 0.100 M NH_4CdI_3 until a complete phase transfer occurred. The resulting QDs in FA were washed with toluene twice, precipitated them with acetonitrile to remove access ligand, and redispersed in formamide.

Characterization Methods

Optical absorption measurements. Absorption spectra of colloidal solutions were collected using a JASCO V780 UV-visible-NIR spectrometer.

Photoluminescence measurements. PL spectra in the wavelength range of 500 – 1500 nm were collected using FluoroMax+ spectrofluoremeter (Horiba Jobin Yvon).

PL QY measurements

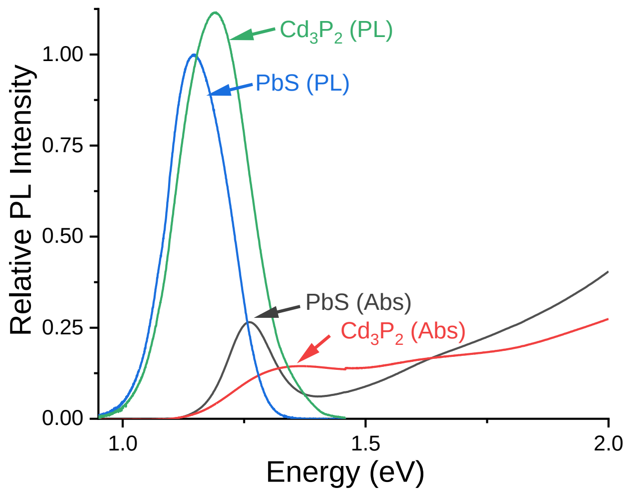

Figure S2. QY measurement of a sample of Cd_3P_2 QDs by comparison with a sample of PbS QDs with the QY of 30%.

Transmission electron microscopy (TEM). The imaging was carried out using 200 kV FEI Tecnai F20 microscope. Samples were prepared by drying 10 – 20 µl of diluted nanocrystal solution in toluene onto a carbon-coated copper grid (Ted Pella).

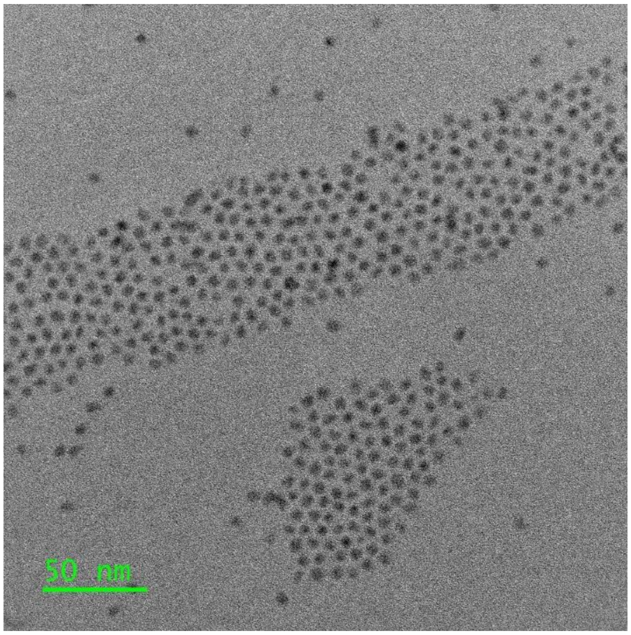

Figure S1. TEM image of 5.8 nm Cd_3P_2 QDs emitting at 1250 nm (the reddest sample in Fig. 1b).

Inductively couple plasma (ICP) optical emission spectroscopy (OES) measurements. About 1 mg of each sample was digested in 0.5 ml concentrate nitric acid (ICP grade) and diluted with deionized water to 10 ml.

Table S1. Atomic compositions of Cd_3P_2 and Cd_3P_2/CdSe QDs measured by ICP OES and referred to P (2.00).

Sample	Cd	P	Se
**Cd_3P_2 QDs (oleate-capped)**	3.38	2.00	<0.01
**Cd_3P_2/CdI_2 QDs**	4.34	2.00	<0.01
**Cd_3P_2/CdSe QDs**	3.63	2.00	0.22

Time-Resolved PL measurements

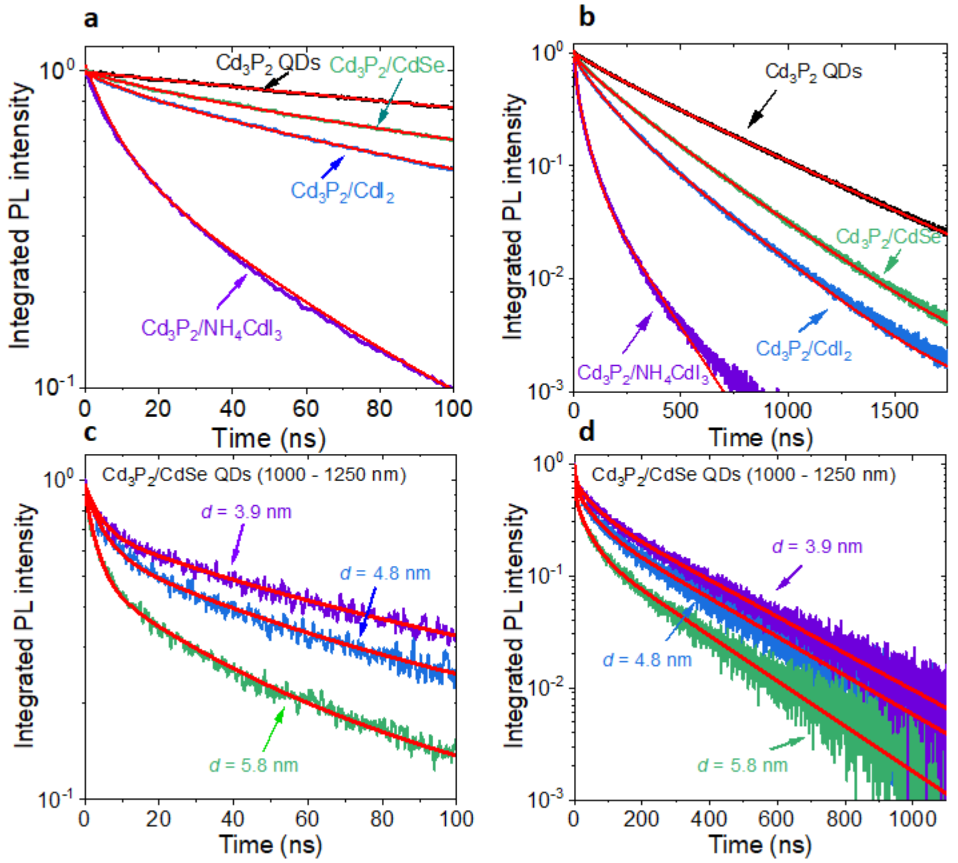

Figure S3. PL dynamics of bare and passivated Cd_3P_2. (a) First 100 ns of time-resolved PL of Cd_3P_2, Cd_3P_2/CdSe, Cd_3P_2/CdI_2, and Cd_3P_2/NH_4CdI_3 QDs excited with a 629 nm pulsed laser diode and emitting ca. 850nm fitted with the minimal number of multiexponentials. (b) Time-resolved PL of Cd_3P_2, Cd_3P_2/CdSe, Cd_3P_2/CdI_2, and Cd_3P_2/NH_4CdI_3 QDs excited with a 629 nm pulsed laser diode and emitting ca. 850 nm fitted with the minimal number of multiexponentials. (c) First 100ns of time-resolved PL of Cd_3P_2/CdSe QDs excited with a 705 nm laser with the emission peaks 1000 – 1250 nm (the QDs shown in Fig. 1b). (d) Time-resolved PL of Cd_3P_2/CdSe QDs excited with a 705 nm laser with the emission peaks 1000 – 1250 nm (the QDs shown in Fig. 1b).
